# Cost-Effective Engineered Cementitious Composites with Hybrid PVA and Basalt/PP Fiber: A Study on Compressive, Tensile and Impact Performance

**DOI:** 10.3390/ma16145172

**Published:** 2023-07-23

**Authors:** Weibin Liao, Peizong Wu, Jiatao Huang, Gai Chen, Jiaxiang Lin, Yongchang Guo, Runsheng Chen

**Affiliations:** School of Civil and Transportation Engineering, Guangdong University of Technology, Guangzhou 510006, China; lwb200111@163.com (W.L.); wupeizong2001@163.com (P.W.); hjt291184@163.com (J.H.); 13113976160@163.com (G.C.); jxiang.lin@gdut.edu.cn (J.L.); c1159135302@163.com (R.C.)

**Keywords:** engineered cementitious composites, hybrid fiber, mechanical performance, impact performance

## Abstract

Currently, oil-coated PVA fibers are the most commonly used material in ECC research. However, the high price limits the application of PVA-ECC in practical engineering. In order to reduce the cost, one of the methods is to partially replace the PVA fibers in ECC. In order to demonstrate the feasibility of PVA/BF-ECC and PVA/PP-ECC, polyvinyl alcohol fibers (PVA), basalt fibers (BFs) and polypropylene fibers (PP) were added at 0.5%, 1.0% and 1.5% by volume of PVA in addition to 1% by volume of PVA. Subsequently, tensile, compression and drop-weight impact tests were conducted on single or hybrid fiber concrete. The results showed that the post-peak compression toughness, tensile strength, and initial cracking impact strength of PVA/BF-ECC and PVA/PP-ECC increased significantly with the increase in the volume ratio of BF and PP fibers, while the performance of PVA-ECC materials with the same fiber volume ratio decreased slightly. Therefore, the cost can be reduced by designing hybrid PVA/BF-ECC materials that meet the performance requirements. The experimental evidence presented in this study demonstrates the feasibility and reasonable prospect of the new hybrid PVA/BF-ECC.

## 1. Introduction

Concrete is an important construction material widely used in building construction [1]. However, the tensile strength of ordinary concrete is relatively low and is prone to cracking and damage, especially when subjected to shock and vibration [2]. Therefore, in order to improve the tensile strength and toughness of concrete, the addition of fibers is now widely used to improve the performance of concrete [3,4], thus improving the fracture toughness of concrete [5,6,7]. In this regard, researchers have pioneered the study of cementitious materials with certain fibers [8,9,10,11] and have achieved successful applications.

As the mechanical properties of concrete materials continue to be explored, Victor Li [12] has created a concrete material that is capable of tensile load-bearing capacity after initial cracking and exhibits strain-hardening and multiple cracking behavior through the unique design of fibers, matrix and fiber/matrix interface, which is known as engineered cementitious composite (ECC). Currently, most of the studies on ECC are based on the addition of PVA. The results show that the tensile strain of PVA-ECC can reach 1–9% [13,14,15], and the average dense crack width can be controlled to about 60 microns when the strain exceeds 1% [16,17,18]. With the addition of PVA, ECC is more ductile than mortar under uniaxial compression and exhibits a ductile shear breakage mode rather than brittle splitting [19,20,21]. Zhang Zhigang et al. showed that the addition of PVA was sufficient to change the failure mode of cementitious composites from brittle to ductile, with excellent energy absorption and impact toughness. Compared with ordinary concrete, ECC has better mechanical properties and can better meet the needs of modern civil engineering [22]. However, the fibers currently used are generally indicated as Oiled Polyvinyl Alcohol Fiber (PVA) [23], which is monopolized by Kuraray in Japan, and its high price limits the application of ECC in practical engineering [24]. Therefore, the development of lower-cost ECC materials is an important issue to promote their application in practical engineering.

One way to reduce the cost of PVA-ECC is to partially replace the high-cost oiled PVA with relatively low-cost fibers. It has been shown that blending PVA fibers with PET fibers [25], PP fibers [26,27], steel fibers [28,29], and POM fibers [30] can effectively reduce the cost. Wang et al. [31,32] showed that the incorporation of recycled steel fibers or short-cut steel wool fibers could improve the compression and tensile strengths but reduce the tensile strain capacity. Maalej et al. [33] found that blending steel-PE fibers could improve the tensile properties of ECC. The results showed that blending BF-PVA fibers could significantly improve the compression resistance of ECC and reduce the cost.

The above studies have shown that the incorporation of different types of fibers into concrete can reduce the cost of PVA-ECC while maintaining its mechanical properties. However, different types of fibers have different reinforcing effects on concrete, so mixing different types of fibers in certain proportions allows them to perform their respective advantages at different stages and levels. In addition, the effects of mixing them with PVA fibers have not been adequately studied. Therefore, the effect of different ratios of PVA/BF-ECC and PVA/PP-ECC on the mechanical properties (e.g., compression, tensile, and impact resistance) of the concrete is not known.

In order to investigate the effects of BF and PP fibers on the mechanical properties of concrete, the hybrid PVA/BF-ECC, PVA/PP-ECC and pure PVA-ECC were tested via axial tensile, axial compression and drop-weight impact test. It is shown that the hybrid BF and PP fibers can significantly improve the compression, tensile and impact properties of concrete. In addition, the effects of different blending ratios on the performance of concrete were compared and analyzed to find out which hybrid fibers are more effective in improving the mechanical properties of concrete.

## 2. Experimental Program

### 2.1. Materials and Mixing Ratios

**Figure 1:**
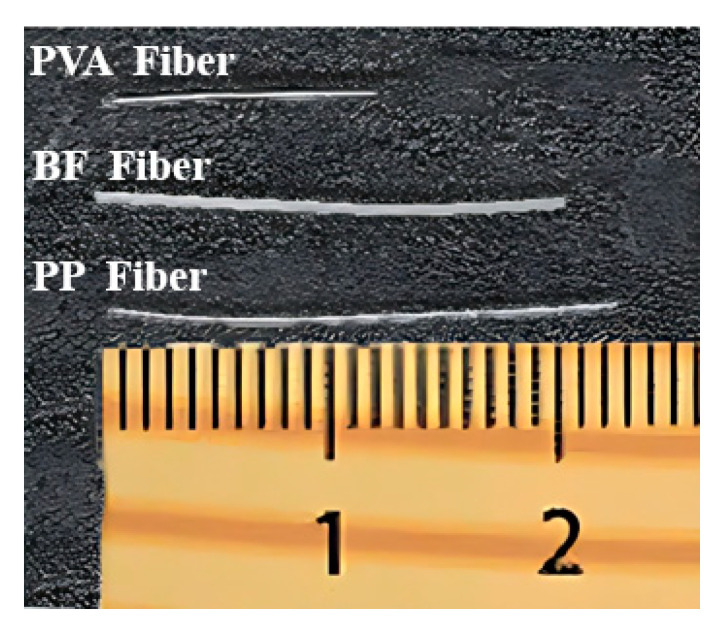
PVA Fiber and BF Fiber.

The test materials used in this experiment included silicate cement (PII52.5R), fly ash type F (ASTM-C618, 2012), fine silica sand, polycarboxylic acid water reducing agent, water, and fibers (PVA fiber, BF fiber, and PP fiber). Among them, the size range of fine silica sand is 100 to 200 mesh. Ltd., Cq-SHJ09 polycarboxylic acid water reducing agent produced by Shanghai Chenqi Chemical Technology Co, was used. The PP fiber is made of polypropylene fiber produced by Huixiang Fiber Factory. These are shown in Figure 1. The detailed properties of PVA fiber, BF fiber and PP fiber provided by the manufacturer can be seen in Table 1.

In this study, ECC was formulated as shown in Table 2 and divided into 11 groups covering 11 mixing ratios, based on the typical ECC mix (ECC-M45) designed by Li et al. [34] and adjusted to ensure good compatibility of the fresh slurry. To fully investigate the effect of fiber content (volume ratio) on the mechanical properties of ECC, the fiber content was set from 0% to 2.5%. Three groups of PVA/BF-ECC, three groups of PVA/PP-ECC, four groups of PVA-ECC and one group of Plain Cementitious Composite (PCC) were studied. The effects of fiber content of BF/PVA-ECC and PP/PVA-ECC on the mechanical properties of ECC were investigated by comparing the test results of PVA/BF-ECC, PVA/PP-ECC and PVA-ECC, respectively. In addition, the mechanical differences between PVA/BF-ECC, PVA/PP-ECC and PVA-ECC were investigated by comparing the test results of PVA/BF-ECC, PVA/PP-ECC and PVA-ECC with the same fiber volume ratios.

### 2.2. Test Specimens

**Figure 2:**
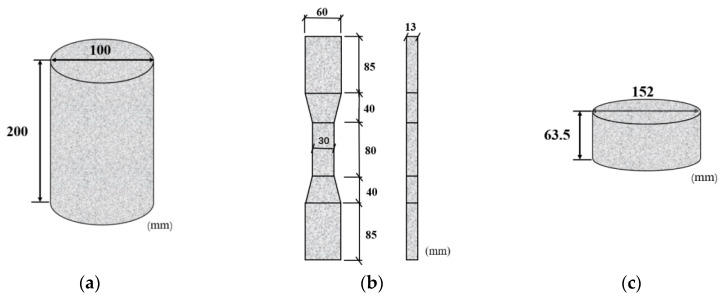
Dimensions of specimens. (**a**) Specimens for axial compression tests. (**b**) Specimen for tensile tests. (**c**) Specimens for drop-weight impact tests.

Axial compression test, axial tensile test and drop weight impact test were carried out. The dimensions of the specimens are shown in Figure 2, and the specimens were referred to Chinese standards (CECS13-2009 [18] JCT2461-2018 [35]). Specifically, a cylindrical specimen with a height of 200 mm and a diameter of 100 mm is shown in Figure 2a for the axial compression test; a dog-bone specimen with a height of 63.5 mm and a diameter of 152 mm is shown in Figure 2b for the axial tensile test Figure 2c. The specimen with a height of 63.5 mm and a diameter of 152 mm was used for the drop-weight impact test, see Figure 2c. A total of 24 specimens were prepared, with each group consisting of three specimens in order to investigate the tensile, compression and impact properties of PVA/BF-ECC, PVA/PP-ECC and PVA-ECC.

The specimens were prepared as follows: first, the cementitious material was added to a 30 L planetary mixer and dry mixed for 3 min. Then, the aggregate was added and dry mixed for another 3 min, after which the accelerator and additional water were mixed and added to the mixer for another 3 min, and the fiber was added while mixing. Finally, the finished paste was poured into the mold, molded, and cured in a standard curing chamber for approximately 24 h and then removed from the mold. The specimens were cured in the curing chamber for 28 days and then demolded and were considered ready for testing.

### 2.3. Test Method

#### 2.3.1. Axial Tensile Test

**Figure 3:**
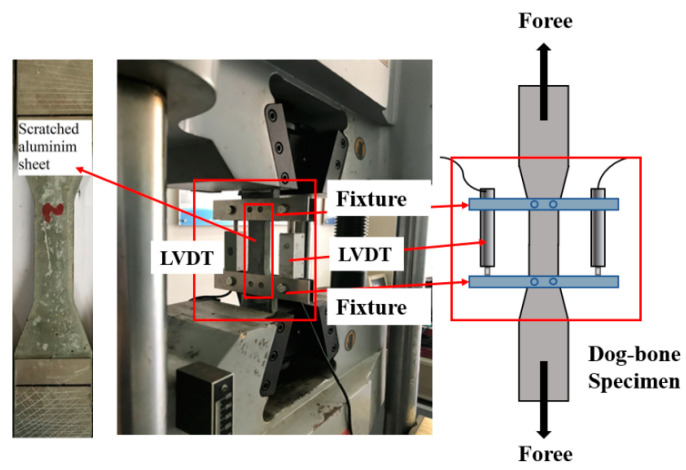
Tensile test set-up.

The axial tensile test was performed using an electronic universal testing machine with the set-up shown in Figure 3. To avoid stress concentration at the end of the specimen during the loading process, aluminum plates were attached to both ends of the tensile specimen to ensure uniform stresses on the specimen. The test specimen was loaded in displacement-controlled mode at a rate of 0.5 mm/min. Linear displacement transducers (LVDTs) with a precision of 1 µm were fixed on both sides of the specimen to observe the deformation of the middle part of the specimen and to determine its tensile behavior, as shown in the figure below. The tensile strain of the specimen was calculated from the average value of the two displacement gauges and the measured length (80 mm), and the tensile stress was calculated from the applied load and the cross-section (30 mm × 13 mm).

#### 2.3.2. Axial Compression Test

**Figure 4:**
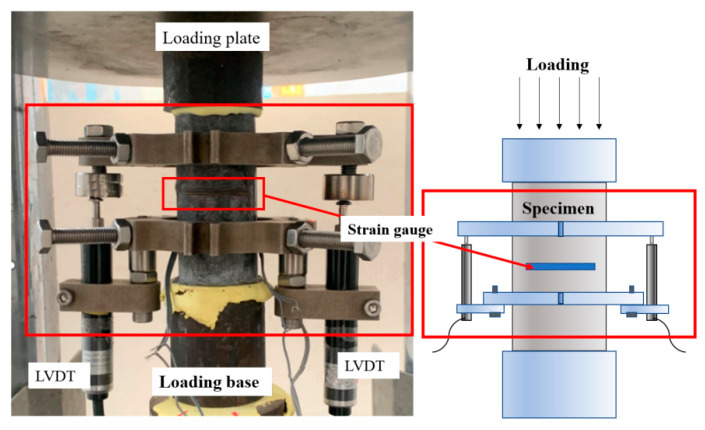
Set-up of the axial compression tests.

The set-up of the axial compression test is shown in Figure 4, referring to the Chinese standard (Standard Test Method for Fiber Reinforced Concrete CECS 13-2009 [36]), and a compression testing machine was used. In order to avoid uneven loading at both ends of the specimen, the specimen needs to be leveled by using high-strength plaster to level the cylindrical section before applying the load. As follows, the force control mode of 0.5 MPa/s was used before the peak condition of the specimen, and the displacement control mode of 0.12 mm/min was used after the peak condition until the specimen failed [36]. During the experiment, the displacement of the specimen was measured simultaneously by strain gauges and LVDTs. In the center of the cylinder, strain gauges of 100 mm and 80 mm in length were glued to measure the axial strain and the circumferential strain, respectively. In addition, two linear displacement gauges were used to measure the axial deformation of the specimens in order to avoid damage to the strain gauges due to concrete cracking and deformation at the beginning of the test. The load, displacement and strain measurements were collected simultaneously by the TDS530 static data acquisition instrument.

#### 2.3.3. Drop-Weight Impact Test

**Figure 5:**
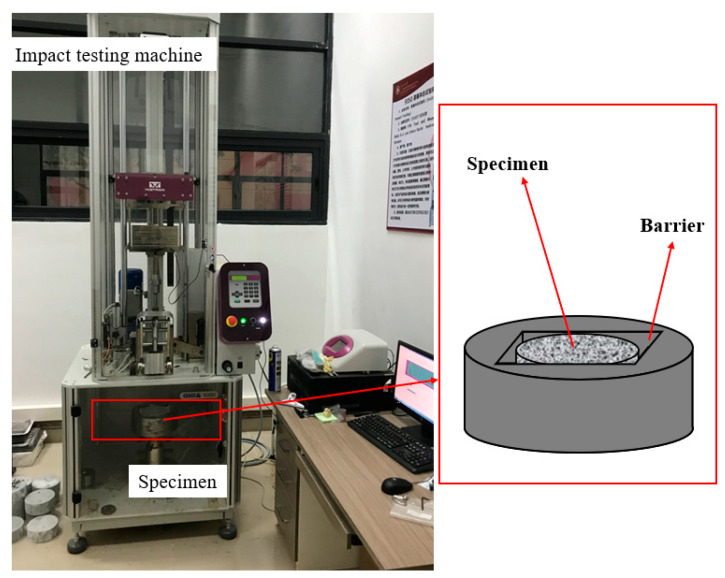
Drop-weight impact test set-up.

The drop-weight impact test was performed according to the method recommended by the ACI 544 committee [37]. The Engstrom CEAST9350 (Figure 5) was used as the impact testing machine. A steel hammer weighing 10.24 kg was dropped 350 mm above the specimen, which provided approximately 35.1 J of energy, and struck the center of the specimen. After each impact, the specimen was carefully observed for cracks or damage, the number of first crack impacts N1, the number of damage impacts N2, and the failure mode of the specimen was carefully recorded. Note that, according to the ACI 544 committee, the damage of the specimen was due to the toughening of the specimen to the barrier layer on any three sides [37].

## 3. Experimental Results and Discussion

### 3.1. Failure Mode

#### 3.1.1. Tensile Test

By observing the fracture process of the specimens, it was found that no cracks appeared on the surface of the specimens at the beginning of the test, but with the gradual increase in load, both PCC and ECC specimens started to crack and formed the first crack. When the number of cracks reached a certain level, the micro-cracks also ceased to appear, but the micro-cracks already formed began to extend and opened up continuously. The fibers across the cracks are pulled out or broken, and the specimen is broken into two pieces and cannot continue to carry the load. After unloading, many of the previously created fine cracks start to shrink due to the action of the fibers, and after removing the specimen, many of the previous fine cracks are difficult to observe and need to be observed with the aid of a microscope.

**Figure 6:**
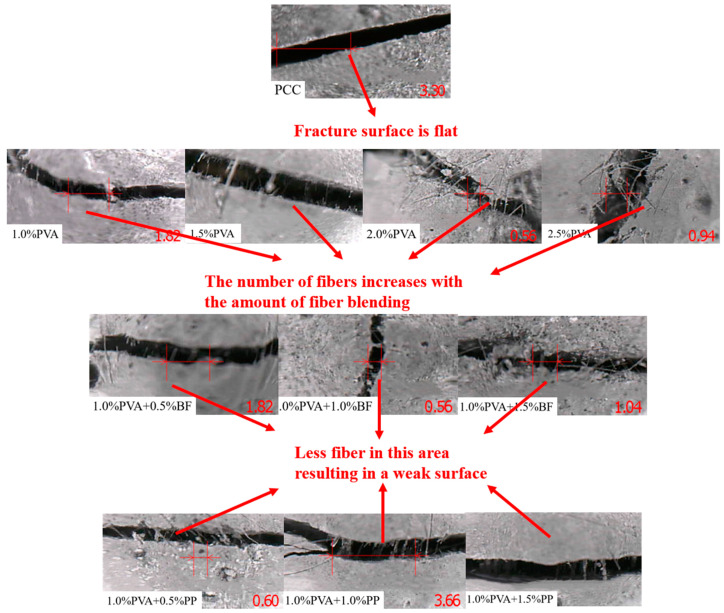
Fracture interface of tensile specimens. Note: The letters in the specimen number indicate the fiber type, A represents PVA, P represents PP, and B represents BF; The number indicates the fiber volume content, such as A-1.0P-0.5 means that the PVA fiber volume content of the specimen is 1.0%, and the volume content of PP fiber is 0.5%.

The typical local fracture characteristics of the specimens can be obtained by observing the local crack patterns of the specimens in Figure 6. The fracture surface of PCC specimens is flat; for PVA-ECC specimens, the number of fibers across the crack increases per unit area with the increase in fiber content; for PVA/BF-ECC specimens, the number of fibers per unit area does not differ much with the increase in fiber content at the crack interface, and the same is true for PVA/PP-ECC. This is because the interface of PVA to the cement matrix is changed due to the incorporation of BF and PP, which causes the fibers to be unevenly dispersed within the matrix and creates a weak surface. Therefore, the number of fibers on the weak side of the crack is not much different between the three specimens with BF and the three specimens with PP.

#### 3.1.2. Compression Test

**Figure 7:**
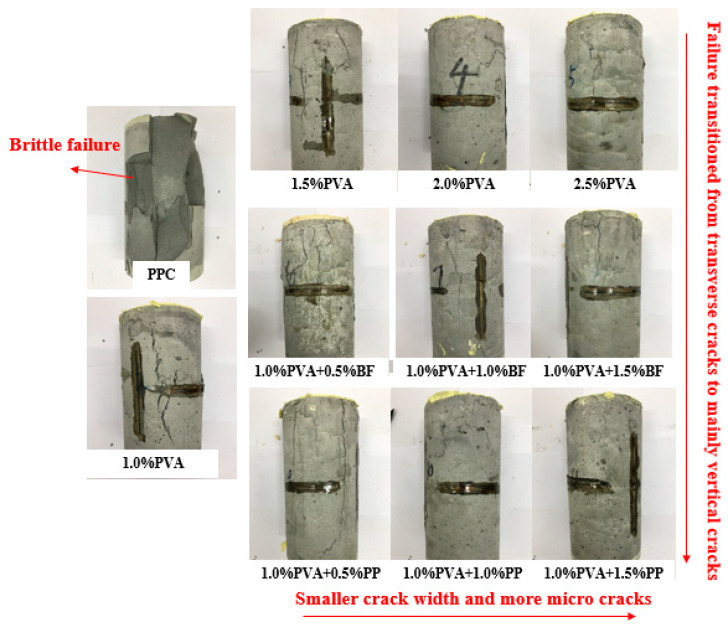
Failure modes of cylinder specimens.

Figure 7 shows the typical damage morphology of the specimens with different fiber content and different types of fibers in the axial compression test. The damage morphology of the first group of PCC matrix specimens is significantly different compared to the other specimens. During compression, cracks in the matrix specimen develop from both ends toward the center. When loaded to the peak load, the specimens eventually showed a brittle failure and split into pieces. In contrast, the hybrid-fiber specimens exhibited multiple cracking characteristics as well as ductility. As can be seen in Figure 7, the crack widths decrease with increasing fiber content, mainly because a certain amount of fiber can form a bridge-like support grid structure inside the concrete, which prevents crack expansion and thus improves the ductility and toughness of the concrete.

In addition, in the hybrid PVA/PP-ECC and hybrid PVA/BF-ECC, the damaged surfaces of the specimens were mainly cracked vertically. In PVA-ECC, the specimens had more transverse cracks on the damaged surface than the other two groups. This is mainly due to the fact that when the cylindrical specimens are compressed in the longitudinal direction, the transverse expansion is inevitable, which generates tensile stresses and forces the material to be stretched. The transverse expansion also generates an outward tensile stress. When this tensile stress exceeds the tensile strength of the specimen, it will crack and produce vertical cracks. If the tensile stress is less than the tensile strength of the specimen, inclined cracking occurs first. When the tensile stress exceeds the tensile strength, vertical cracks will continue to occur. Therefore, it can be concluded that the incorporation of PVA has the most significant improvement in the tensile strength of the material, which is better than BF and PP.

#### 3.1.3. Drop-Weight Impact Test

**Figure 8:**
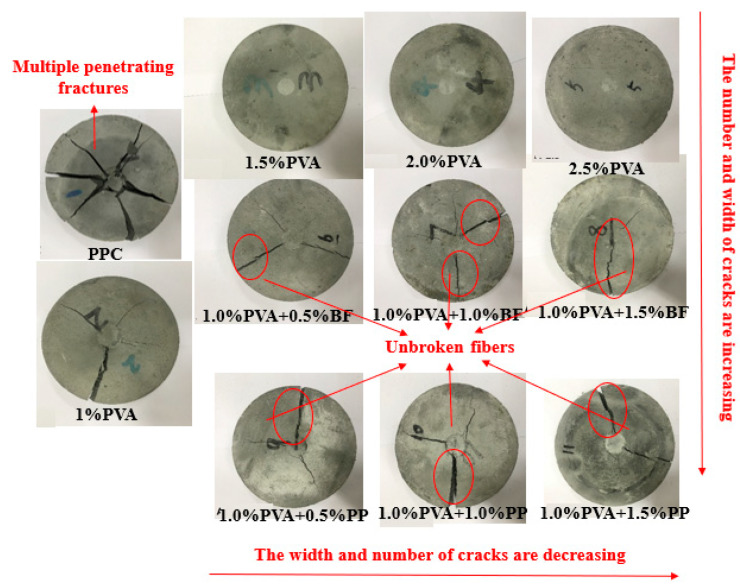
Typical failure modes of impact specimens.

Figure 8 shows the failure mode of different groups of specimens in the drop-weight impact test. It can be seen that after the failure mode, the PCC matrix specimens showed multiple penetration cracks and fragmented into multiple pieces, showing a brittle failure mode. In contrast, the hybrid-fiber PVA-ECC, PVA/BF-ECC and PVA/PP-ECC specimens maintained good integrity after the damage. The cracks were radiated outward from the central impact point, and a large number of fibers could be observed in the cracks. These specimens exhibited a failure mode of cracking without disintegration and fracturing without fragmentation. The reason for this is that the added fibers form a support grid structure in the concrete similar to that of a bridge, and when the specimens are subjected to impact loads, the fibers in the matrix are able to withstand part of the load and slow down the process of concrete damage.

In addition, PVA-ECC specimens showed almost no large cracks compared to PVA/BF-ECC and PVA/PP-ECC specimens at the same fiber volume ratio. This is due to the higher tensile strength and stiffness of the PVA, which provide greater restraint to the matrix and result in better impact performance of PVA-ECC. Specifically, the PVA-ECC specimen showed only one penetrating column crack and several micro-cracks, and there were still some unbroken PVA between the main cracks. In comparison with the PVA/BF-ECC and PVA/PP-ECC specimens, the latter had more main cracks and larger crack widths, which resulted in a poorer ability of the specimens to absorb impact energy. This is due to the fact that the tensile strength of PP is lower than that of BF, and most of the fibers are pulled out early when the specimens are subjected to the impact load, resulting in a lack of resistance to the further expansion of the main crack, and therefore the width of the main crack is wider than that of the other groups.

### 3.2. Effect of Hybrid Fibers on Tensile Behavior

#### 3.2.1. Axial Tensile Stress–Strain Curve

**Figure 9:**
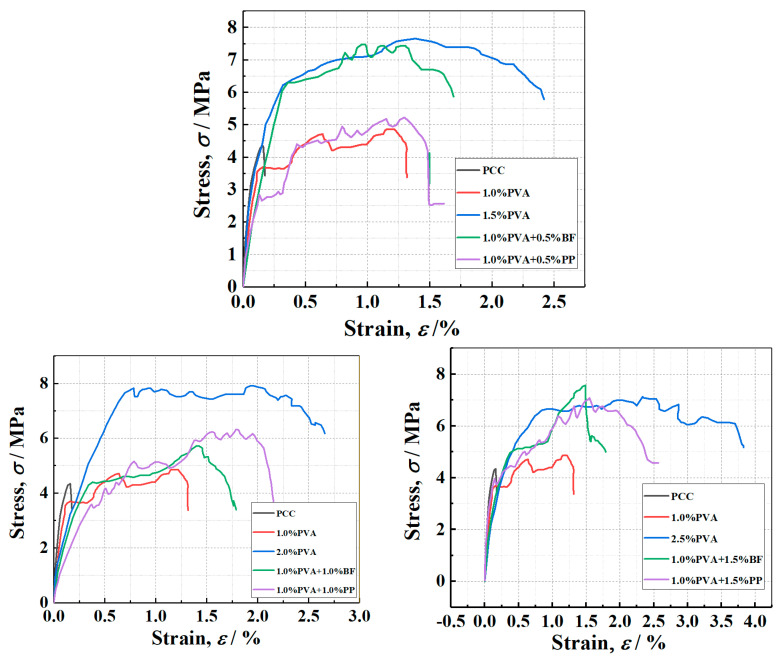
Typical tensile stress-strain curves for PVAECC, PVA/BF-ECC, and PVA/PP-ECC.

Figure 9 shows the tensile stress–strain curves of PVA-ECC specimens as well as PVA/BF-ECC and PVA/PP-ECC specimens. It can be seen from Figure 9 that, except for the PCC specimen, all the hybrid-fiber specimens showed significant strain hardening, and the best tensile performance of PVA-ECC was achieved at the PVA volume ratio of about 2%.

The rate of tensile strain hardening was significantly higher for PVA/BF-ECC and PVA/PP-ECC than for PVA-ECC, but the reasons for this phenomenon were different for these two types of concrete. For PVA/BF ECC, due to the high density of BF, the tensile stress in the specimen decreases rapidly due to the absence of fiber bridging force after the weak surface is pulled off. The tensile stress also decreases rapidly after fiber pullout.

#### 3.2.2. Effect of Hybrid Fibers on Tensile Properties

**Figure 10:**
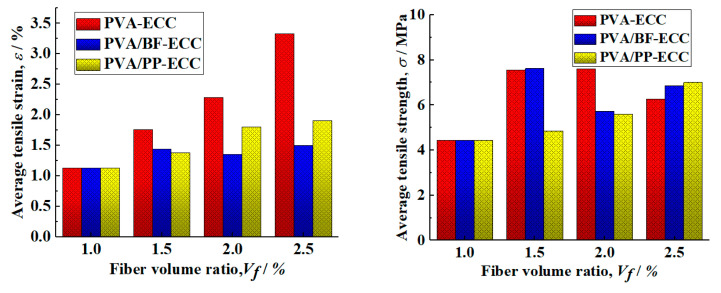
Relationship between average tensile strength and ultimate tensile strain with volume fraction for PVA-ECC, hybrid PVA/BF-ECC, and hybrid PVA/PP-ECC.

Table 3 shows the average tensile strength and ultimate tensile strain of the specimen. The tensile strength and ultimate tensile strain of PVA-ECC, PVA/BF-ECC and PVA/PP-ECC specimens of different kinds of fibers under the same fiber volume content are shown in the table. At the same time, Figure 10 also shows the tensile strength and ultimate tensile strain of these specimens.

According to the results of Figure 10, the tensile strength and ultimate tensile strain of the PVA-ECC specimen increase with the increase in the PVA volume ratio, while the tensile strength and ultimate tensile strain of the PVA/PP-ECC specimen (PVA volume ratio of 1%) also increase with the increase in the PP volume ratio. This is due to the good compatibility of PVA and PP with the matrix. When BF was added to PVA/BF-ECC (PVA volume ratio of 1%), the tensile strength of the specimens decreased with the increase in BF volume ratio and the ultimate tensile strain was close. This is due to the high density of BF, which leads to the incorporation of more mass of BF in the same volume of admixture and reduces the fluidity of the cement mortar, and also due to the fact that the basalt fibers are less compatible with the matrix in the mix than the other two fibers. The fibers tend to agglomerate and disperse unevenly, which leads to weak areas at the interface of the specimen. The number of fibers in the cross-section of the weak zone is low, and cracks will preferentially develop in the weak zone during the tensile test, resulting in lower tensile strengths and close ultimate tensile strains in the specimens. For a given fiber volume ratio, the ultimate tensile strains of PVA/BF-ECC and PVA/PP-ECC were slightly lower than those of PVA-ECC, and the tensile strength of PVA-ECC was improved more significantly by BF than by PP.

### 3.3. Effect of Hybrid Fibers on Compression Behavior

#### 3.3.1. Axial Compression Stress–Strain Curve

**Figure 11:**
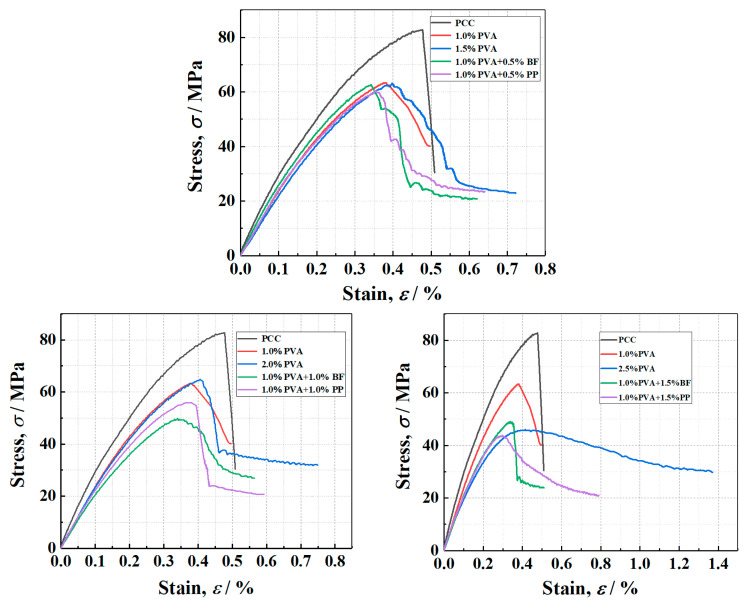
The stress–strain curves of each group of cylinders under compression.

The typical axial stress–strain curves of PVA-ECC specimens, PVA/PP-ECC specimens and PVA/BF-ECC specimens is shown in Figure 11. As can be seen from Figure 11, the compressive strength of all ECC specimens is generally lower than that of PCC specimens, which indicates that the addition of fibers has a weakening effect on the compression strength of concrete. On the basis of 1% by volume of PVA fiber, the specimens with BF and PP showed a decrease in compression strength with the increase in fiber content. After the intensity reaches its peak, the specimens showed a rapid decrease, which is similar to the results of the existing PVA-ECC [38,39] studies.

However, the specimens doped with PVA alone showed the opposite performance; with a range of increasing fiber volume ratio, the compression strength increased slightly. This is due to the dual effect of weakening and strengthening of the fiber on the compression strength of the material. On the other hand, after the cracking of the matrix, the specimen is mainly loaded by the fibers across the crack. With an increase in fiber dosing, the number of fibers per unit area at the crack interface of the specimen also increases. As the crack develops further, more fibers can absorb additional energy, leading to greater deformation in the specimen and a relatively larger residual strain. This results in improved energy absorption capabilities and resistance to damage.

Overall, although the addition of fibers slightly weakened the compression strength of ECC, it significantly improved the integrity of the specimens after compression damage and also inhibited the expansion of crack width.

#### 3.3.2. Influence of Hybrid Fiber on Compression Strength and Elastic Modulus

**Figure 12:**
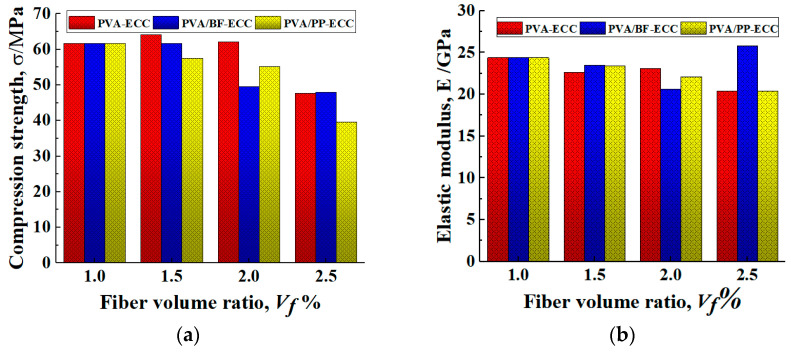
Compression strengths and elastic modulus of PVA-ECC and PVA/PP-ECC with different fiber volume ratios. (**a**) Compression strength. (**b**) Elastic modulus, E.

In order to observe the effect of fiber content of PP and BF on the axial compression behavior of PVA-ECC and to compare the differences between PVA/PP-ECC, PVA/BF-ECC and PVA-ECC with the same fiber volume ratio, the compression strength and elastic modulus of PVA/PP-ECC, PVA/BF-ECC and PVA-ECC specimens are shown in Figure 12. The compression strength was obtained directly from the stress–strain curves of the specimens, while the modulus of elasticity was calculated according to the specifications in ASTM C469/C469 M [40].

It was observed that the compression strength of PVA/BF-ECC and PVA/PP-ECC decreased gradually with the increase in fiber volume ratio, while the compression strength of PVA-ECC changed slightly and showed an increasing trend when the volume ratio increased from 1.0% to 1.5%. This can be attributed to the fact that with the increase in the volume ratio of fibers, the probability of their formation in the concrete has increased, thus weakening the compression strength of the concrete to some extent. In addition, it was found that when the volume ratio of PVA in the specimens was constant at 1%, the compression strength of the specimens decreased by 6.73%, 10.6%, and 35.7% when additional 0.5%, 1.0%, and 1.5% PP were added to the specimens, respectively. For the BF specimens, the compression strengths decreased by 0.02%, 19.60% and 3.27%, respectively. Therefore, the compression strength of PVA/PP-ECC is more sensitive to the fiber content.

Figure 12 also shows that the elastic modulus of the hybrid-fiber specimens is lower than that of the specimens without fiber. This indicates that the fiber incorporation significantly reduces the stiffness of the specimens and increases the probability of deformation of the specimens, thus increasing their flexibility. On the basis of 1%PVA fiber volume ratio, the elastic modulus increased significantly at 1.5% BF fiber volume ratio. This is because when the BF reaches 1.5%, the internal BFs have a large amount of agglomeration, which makes the proportion of effective working fibers in the specimen greatly reduced. The difference in elastic modulus of the other fiber volume ratio specimens was not significant. Therefore, the fiber volume ratio has a major effect on the elastic modulus of the specimens, while the type of fibers has a minor effect.

#### 3.3.3. Poisson’s Ratio

Poisson’s ratio is an important mechanical parameter of the material, which is defined as the ratio of the absolute value of the transverse positive strain to the axial positive strain when the material is subjected to unidirectional tensile or compression and is also known as the transverse deformation coefficient. Poisson’s ratio reflects the relationship between longitudinal shrinkage and transverse deformation of the material under compression and is also an important basis for the variation of the elastic range of concrete. In this experiment, the Poisson’s ratio was calculated by using Equation (1) specified in ASTM C469/C469M: S_1_ represents the load at which the longitudinal strain of the specimen reaches 0.000050, while S_2_ corresponds to 40% of the ultimate load of the specimen. It is worth noting that the strain value at 40% of the ultimate load was determined through interpolation.

µ = (ε_t2_ − ε_t1_)/(ε_2_ − 0.00050) (1)

where: 

µ—Poisson’s ratio

ε_t2_—lateral strain caused by stress S_2;_

ε_t1_—lateral strain caused by stress S_1;_

ε_2_—axial strain caused by stress S_2_.

**Figure 13:**
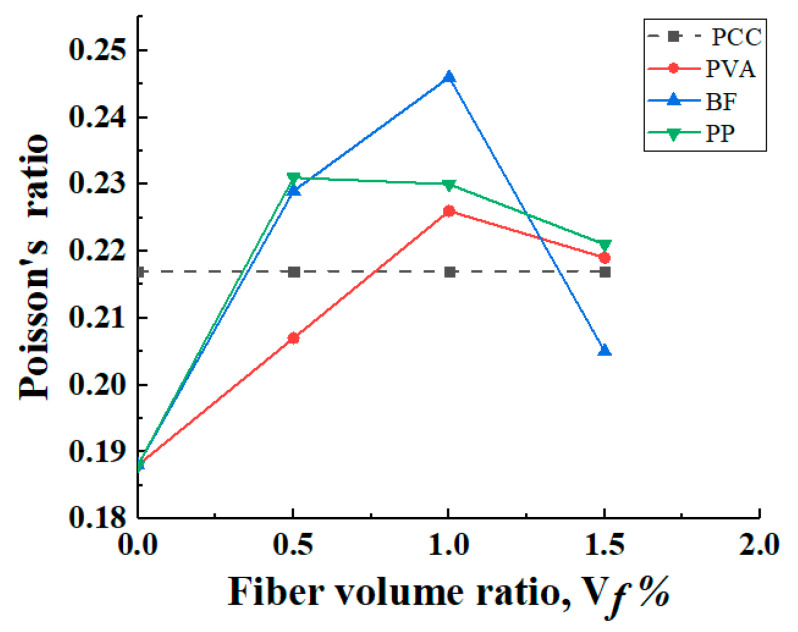
Poisson’s ratio.

According to Figure 13, the Poisson’s ratio of most of the hybrid-fiber specimens is significantly higher than that of the non-fiber-reinforced PCC matrix specimens, and the overall range of Poisson’s ratio is between 0.188 and 0.246. In addition, the Poisson’s ratio of specimens with the same fiber type showed a trend of increasing and then decreasing, indicating that there is an optimal fiber incorporation to achieve the maximum Poisson’s ratio of the specimens. This is because, at low fiber content, the fiber cannot fully utilize its reinforcement effect, resulting in a limited impact on the Poisson’s ratio. At high fiber content, excessive mutual interference between fibers may occur, which may cause local aggregation inside the concrete. The fiber volume content is the best amount between 0.25 and 1.25; when the fiber distribution is uniform, the fiber evenly shares the stress in the concrete, increases the stiffness and strength of the concrete, and improves the ratio between longitudinal and transverse strain, thereby affecting the Poisson’s ratio and making the Poisson’s ratio reach the maximum. The results reveal the potential of adding fibers to improve the mechanical properties of cement composites, and the mechanical behavior can be optimized by adjusting the fiber admixture.

### 3.4. Effect of Hybrid Fibers on Impact Behavior

#### 3.4.1. Effect of Hybrid Fiber Content on Impact Strength

Table 4 shows the results of drop hammer impact tests for PVA-ECC, PVA/BF-ECC and PVA/PP-ECC with different fiber content. The impact ductility coefficient β is calculated by Equation (2), which reflects the energy dissipation and deformation capacity of concrete after matrix cracking and is of great importance for the safety of concrete [41].

β = (N_2_ − N_1_)/N_1_ (2)

where: 

β—Ductility coefficient of the specimen;

N_1_—Number of impacts at the first crack of the specimen;

N_2_—The number of impacts when the specimen is damaged.

The experimental results showed that the addition of BF and PP could effectively improve the impact resistance of PVA/BF-ECC, and the impact resistance of PVA/BF-ECC and PVA/PP-ECC increased with the increase in fiber content. For example, the initial impact strength of PVA/BF-ECC increased by 160%, 440% and 540%, respectively, and the impact strength increased by 150%, 217% and 283%, respectively, when BF and PP with 0.5%, 1.0% and 1.5% volumetric content were mixed into PVA-ECC with 1% volumetric content of PVA. The initial splitting impact strength of PVA/PP-ECC increased by 80%, 180% and 240%, respectively, and the impact strength increased by 100%, 133% and 192%, respectively. Therefore, whether it is BF fiber or PP fiber, the effect of improving the initial cracking impact strength of concrete is better than that of improving the impact strength, which is mainly due to the fact that before the concrete cracks, the fibers form a fine mesh structure inside the material to bear the load together, while after the concrete cracks, some of the fibers withdraw from the work due to fracture or pull out and do not continue to bear the load.

**Table 4:** Drop-weight impact test results.

Groups	First Crack Blows, N1	Failure Blows, N2	First-Crack Impact Energy, E1/J	Failure Impact Energy, E2/J	Ductility Factor, β
PCC	1	1	35.12	35.12	0
1.0%PVA	5	12	175.6	421.44	1.4
1.5%PVA	26	61	913.12	2142.32	1.35
2.0%PVA	141	314	4951.92	11,027.68	1.23
2.5%PVA	234	477	8218.08	16,752.24	1.04
1.0%PVA + 0.5%BF	13	30	456.56	1053.6	1.31
1.0%PVA + 1.0%BF	27	38	948.24	1334.56	0.41
1.0%PVA + 1.5%BF	32	46	1123.84	1615.52	0.44
1.0%PVA + 0.5%PP	9	24	316.08	842.88	1.67
1.0%PVA + 1.0%PP	14	28	491.68	983.36	1.41
1.0%PVA + 1.5%PP	17	35	597.04	1229.2	1.23

#### 3.4.2. Effect of Fiber Type on Impact Strength and Ductility of Specimens

**Figure 14:**
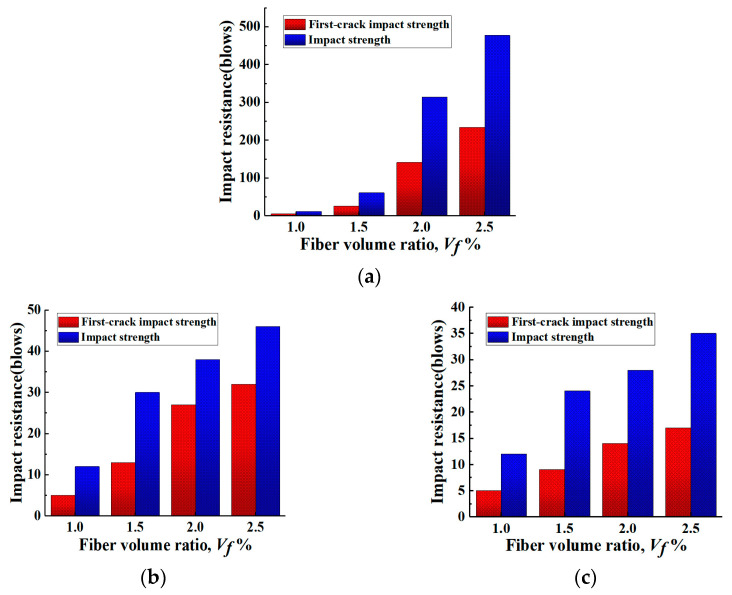
Relationship between impact resistance and fiber volume ratios for PVA-ECC and hybrid PVA/PP-ECC. (**a**) PVA-ECC. (**b**) PVA/BF-ECC. (**c**) PVA/PP-ECC.

Figure 14 shows the experimental results on the number of impacts that the specimens can withstand when PP and BF are added to 1.0% of PVA. It can be found that the number of impacts at first cracking and damage of the specimens with additional PVA content on top of 1.0%PVA fiber content is much higher than that of the specimens with BF fiber and PP fiber content. This is due to the higher density of BF, which reduces the compactness of the specimen matrix and generates more weak zones at higher dosing levels. In the case of PP, the breakage is a pull-out break rather than a pull-out break, resulting in a shorter time for the fibers to bridge, which reduces their energy absorption capacity. Therefore, although both BF and PP can improve the impact resistance of ECC, the improvement is not as pronounced as that of PVA.

From Figure 14, it can be found that on the basis of 1.0% of PVA, the increase in additional PVA from 0.5% to 1.0% has the highest efficiency in improving the number of damage impacts of the specimens; while for the additional PP and BF, the increase in 0.5% in the amount of PP and BF has the highest efficiency in improving the number of damage impacts of the specimens. Therefore, the “power-up period” of different fibers for the improvement of the number of damage impacts was different, with PVA at medium dosing and BF and PP at low dosing.

As outlined in Table 4, it can be found that the improvement of impact resistance of the specimens was significantly different when different types of fibers were mixed with PVA at 1%. The ductility coefficient of the specimen mixed with PVA and PP reached 1.67 at 0.5% of PP, which is the highest among all the specimens, and the ductility coefficient of the specimens mixed with different types of fibers at the same volume is higher than the other specimens. This indicates that the ductility coefficient of the specimens was significantly improved by mixing a small amount of PP with 1.0% of PVA.

## 4. Conclusions

In this paper, the tensile test, compression test and drop-weight impact test of PVA/BF-ECC, PVA/PP-ECC and PVA-ECC were tested via axial tensile, axial compression and drop-weight impact tests, and the effects of BF on PVA/BF-ECC and PP on PVA/PP-ECC were investigated. The results were also compared with the matrix without fiber content, and based on the results and discussions, the following conclusions were drawn:

(1) In the axial tensile test, all specimens generally exhibited strain hardening. During the crack expansion of the specimens, the PVA and BF exhibited a similar failure mode, known as pull-out failure. In contrast, PP demonstrated a fracture failure mode. In the axial compression test, the addition of fibers resulted in a ductile failure mode, which ensured the integrity of the specimens after the damage, and the PVA/PP-ECC specimens showed the most significant inhibition of crack width at the same fiber volume ratio. In the drop-weight impact test, the non-fiber-reinforced specimens showed bursting damage after damage, and the specimens were broken into many pieces, showing obvious brittle damage, while the hybrid-fiber specimens could still maintain good integrity after damage, and the cracks were radiated outward from the central impact point, and a large number of fibers could be observed in the cracks, and the specimens showed the failure mode of cracking but not scattering and breaking.
(2) The tensile strength and ultimate tensile strain of PVA/BF-ECC and PVA/PP-ECC increased with the increase in fiber incorporation. The ultimate tensile strain of PCC is very low, only 0.17%. However, the specimens incorporating fibers exhibit much higher ultimate tensile strains compared to PCC, indicating that the addition of fibers can significantly enhance the material’s ultimate strain. For a given fiber combination, the ultimate tensile strain increases with the fiber content, indicating that increasing the fiber content improves the material’s ultimate tensile strain, with PVA fibers showing the most significant enhancement in the material’s ultimate tensile strain. It is worth noting that the tensile strength of PVA/BF-ECC and PVA/PP-ECC is slightly lower than that of PVA-ECC, which is due to the different mechanisms of action of different fiber materials on concrete.
(3) The effect of fiber content was more obvious than that of fiber type on the compression stress–strain curves of the specimens; with the increase in fiber content, the compression strength of the specimens decreased, but the descending section of the curve was flatter, the deformation of the specimens was larger, the residual strain was relatively larger, and the energy absorption effect and damage resistance of the specimens were better. With an increase in the volume of BF fibers and PP fibers, compared to PVA-ECC with a volume fraction of 1.0%, at high fiber content, both PVA-ECC and PVA/PP-ECC specimens show a significant decrease in strength. However, PVA/BF-ECC specimens demonstrate better stability at high fiber content without a sudden drop in compressive strength. The fiber incorporation can significantly reduce the modulus of elasticity of the specimens, where the effect of fiber incorporation is greater than the effect of fiber type.
(4) With the incorporation of fibers, the damage characteristics of the matrix in the drop-weight impact test were changed, and the value of the specimen load capacity was significantly increased. In terms of fiber type, PVA has the most significant effect on the peak load capacity of the specimen, while BF fiber and PP fiber have similar effects. As the volume of BF and PP fibers increases (with the original PVA volume fraction at 1.0%), the impact strength of PVA/BF-ECC is significantly higher than that of PVA/PP-ECC. However, for a given total fiber volume ratio, the impact strength of PVA-ECC was higher than the other two.

## Figures and Tables

**Table 1:** Properties of PVA and PP fibers.

Fibers	Length	Diameter	Density	Elastic Modulus	Fracture Elongation
	(mm)	(μm)	(g/cm^3^)	(GPa)	(%)
PVA	12	40	1.3	40	6
BF	15	16	2.6	34	3.1
PP	16	20	0.91	4.3	36.4

**Table 2:** Mixture proportions and test groups (kg/m^3^).

Groups	Cement	Fly Ash	Water	Silica Sand	Water Reducing Admixture	PVA	BF	PP
	kg/m^3^	kg/m^3^	kg/m^3^	kg/m^3^	kg/m^3^	vol%	vol%	vol%
PCC	382	890	297	460	18	0	0	0
1.0%PVA	382	890	297	460	18	1	0	0
1.5%PVA	382	890	297	460	18	1.5	0	0
1.0%PVA + 0.5%BF	382	890	297	460	18	1	0.5	0
1.0%PVA + 0.5%PP	382	890	297	460	18	1	0	0.5
2.0%PVA	382	890	297	460	18	2	0	0
1.0%PVA + 1.0%BF	382	890	297	460	18	1	1	0
1.0%PVA + 1.0%PP	382	890	297	460	18	1	0	1
2.5%PVA	382	890	297	460	18	2.5	0	0
1.0%PVA + 1.5%PP	382	890	297	460	18	1	0	1.5
1.0%PVA + 1.5%BF	382	890	297	460	18	1	1.5	0

**Table 3:** Uniaxial tensile test results.

Groups	Tensile Strength	Ultimate Tensile Strain	Average Tensile Strength	Average Ultimate Tensile Strain
	MPa	%	MPa	%
PCC	4.25	0.16	4.34	0.17
	4.43	0.2		
	4.34	0.16		
1.0%PVA	3.53	1.2	4.44	1.13
	4.9	1		
	4.9	1.2		
1.5%PVA	6.65	1.9	7.54	1.76
	8.37	1.94		
	7.6	1.45		
1.0%PVA + 0.5%BF	7.53	1.33	7.62	1.44
	7.42	1.39		
	7.91	1.61		
1.0%PVA + 0.5%PP	3.87	1.43	4.86	1.38
	5.22	1.31		
	5.5	1.39		
2.0%PVA	6.9	2.63	7.60	2.29
	7.9	2		
	8.0	2.23		
1.0%PVA + 1.0%BF	5.9	1.31	5.73	1.35
	5.5	1.33		
	5.78	1.42		
1.0%PVA + 1.0%PP	4.6	1.77	5.6	1.8
	6.1	2		
	6.2	1.61		
2.5%PVA	5.91	3.4	6.27	3.33
	6.8	2.9		
	6.09	3.7		
1.0%PVA + 1.5%BF	5.51	1.52	6.86	1.5
	7.52	1.49		
	7.56	1.48		
1.0%PVA + 1.5%PP	6.5	1.87	7.0	1.91
	6.67	1.9		
	7.71	1.93		
